# Supplementary material for: Sex‐dependent and sex‐independent regulatory systems of size variation in natural populations
Source: Mol Syst Biol. 2019 Nov 26;15(11):e9012. doi: 10.15252/msb.20199012 (PMC6878047; doi:10.15252/msb.20199012)
Supplement: Supplementary file 1 — Appendix [file MSB-15-e9012-s001.pdf]

Appendix

Sex-dependent and sex-independent regulatory systems of size variation in natural populations

Hirokazu Okada, Ryohei Yagi, Vincent Gardeux, Bart Deplancke and Ernst Hafen

This PDF file includes:

Appendix Figs. S1 to S11

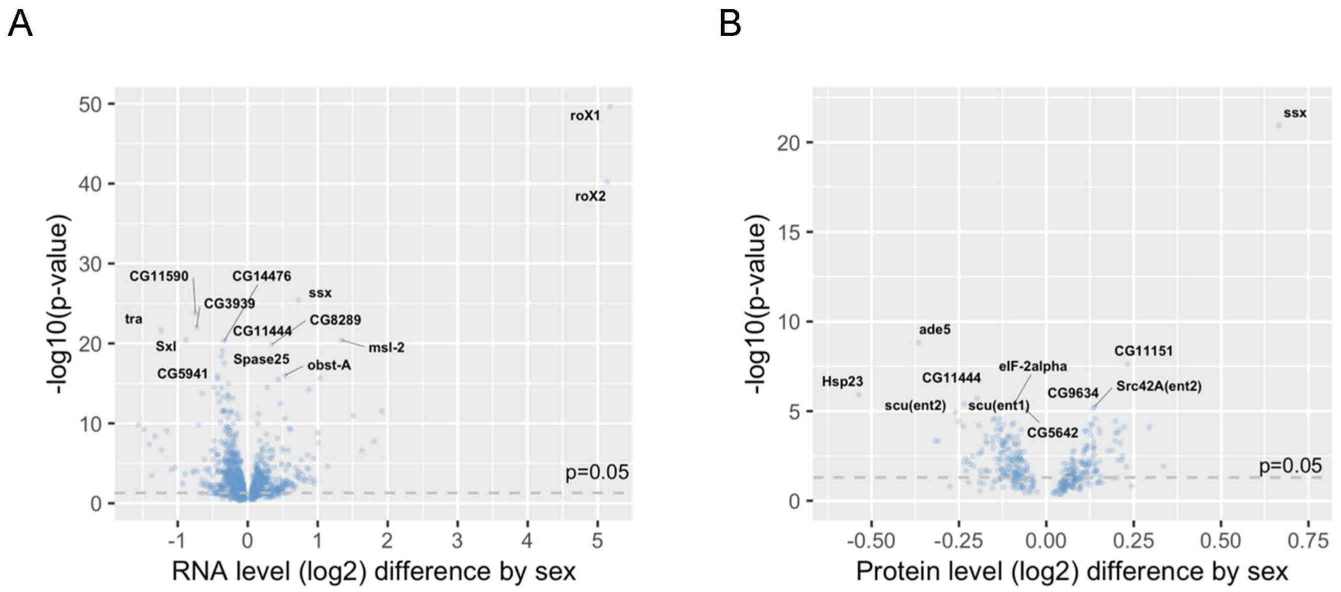

Appendix Figure S1. Most RNAs and proteins with a type 1-exclusive association are differentially expressed between sexes.

Association with sex of RNAs (A) and proteins (B) with a type 1-exclusive association. P-values for the type-3 association are obtained from ANOVA on sex and plotted against expression level difference (male level relative to female level in log2 scale) between sexes. The significance threshold (p=0.05) is indicated by the dashed line. Note that most RNAs and proteins with a type 1-exclusive association are significantly associated with sex.

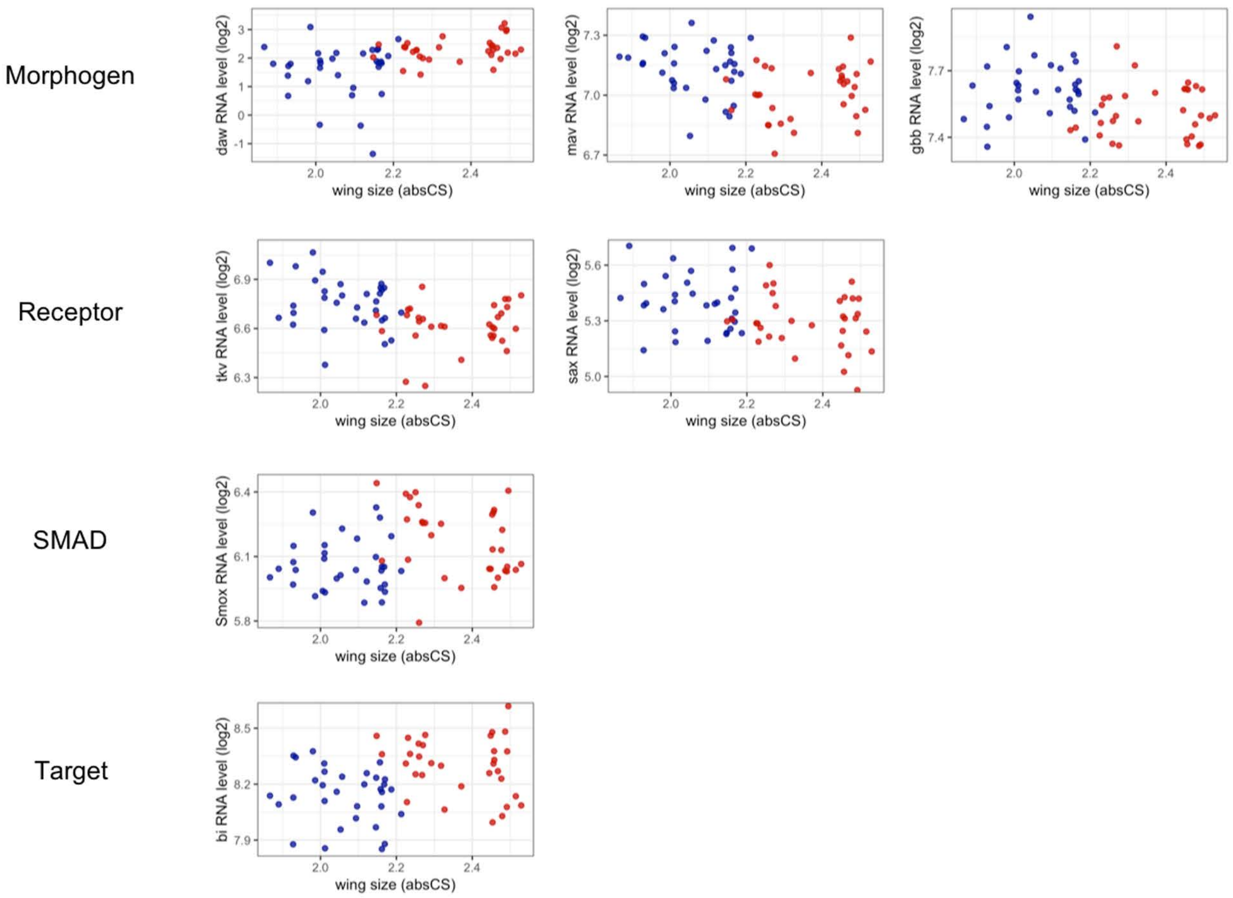

Appendix Figure S2. Pathway components of TGFβ signaling are associated with sexual size dimorphism.

RNA levels of TGFβ signaling pathway components including morphogens, receptors, a SMAD and a target are plotted against wing size (absCS). Note that the RNA levels are significantly different between sexes but not between small and large wing lines within each sex.

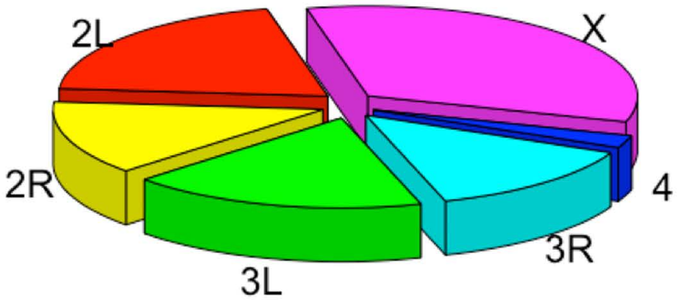

Appendix Figure S3. Canonical growth regulators associated with sexual size dimorphism are spread throughout the genome.
Venn diagram shows that canonical growth regulators associated with wing size via type-3 association are located spread throughout the genome.

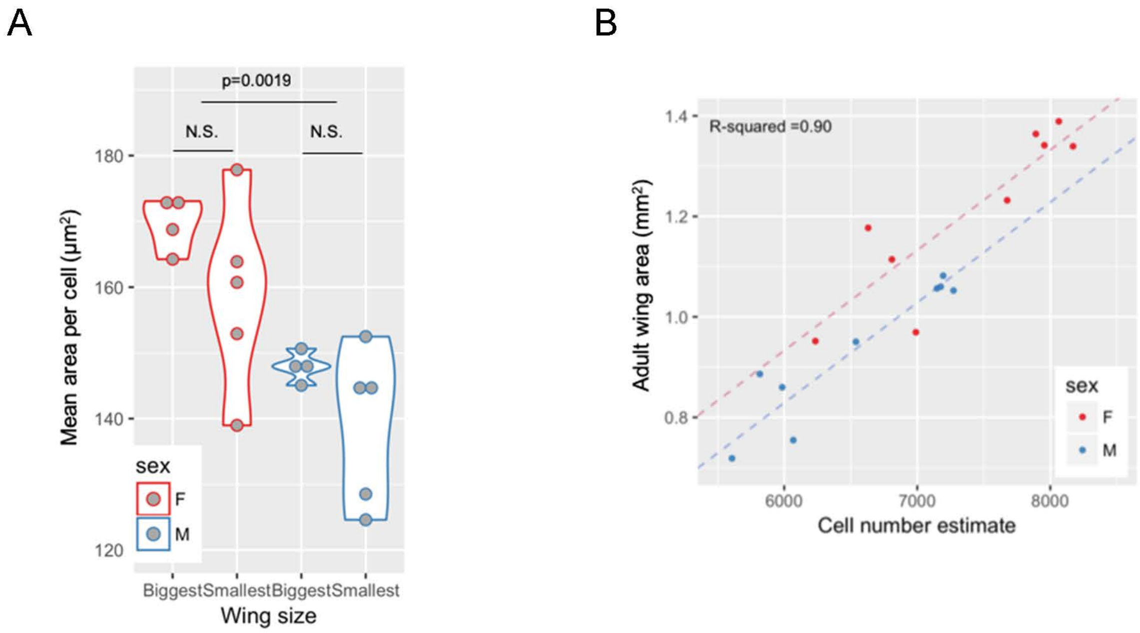

Appendix Figure S4. Association of cell size and number in the wing with wing size.
(A) Sexual dimorphism of cell size in the wing. Cell size is significantly different between sexes but not between the 5 smallest and the 4 biggest wing lines within each sex. Statistical significance was evaluated by Wilcoxon test. (B) Cell number in the wing is proportional to wing area within each sex. The cell number is higher in females, in addition to the larger cell size. The fitted lines by ANCOVA are depicted ($R^2 = 0.9$).

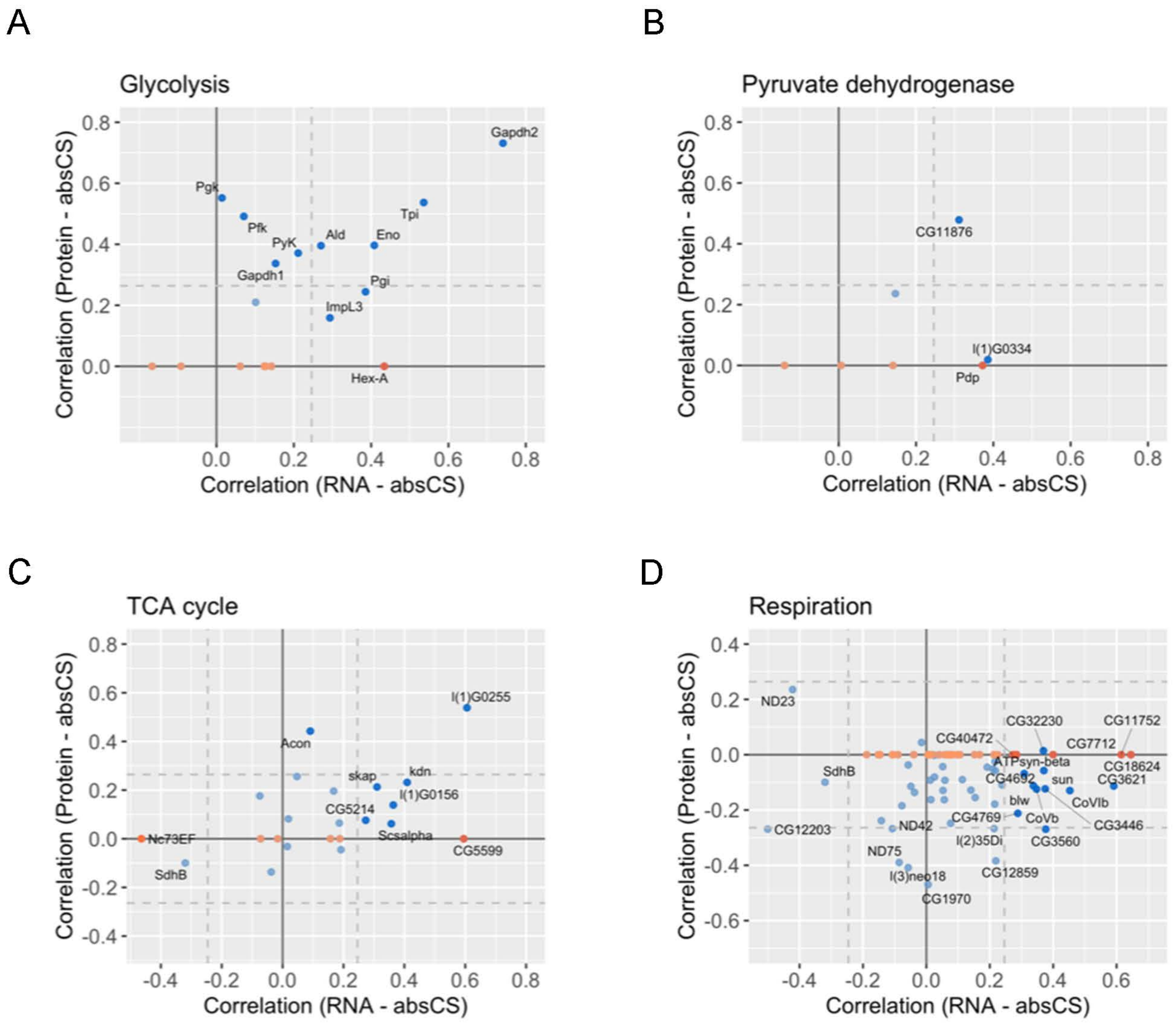

Appendix Figure S5. Glucose metabolism subprocesses associated with wing size variation.
(A-D) Association of glucose metabolism subprocesses (A: glycolysis, B: pyruvate dehydrogenase, C: TCA cycle, D: mitochondrial respiration) with wing size. Spearman correlation coefficients between gene expression (x-axis: RNA and y-axis: protein) and wing size (absCS) are plotted. The genes quantified at RNA level only are indicated by orange dots on the x-axis. The first 3 subprocesses (A, B, C) show positive correlations between both expression levels and wing size. However, in the last subprocess (D), protein levels show negative correlations while RNA levels exhibit positive correlations, suggesting a "Warburg effect" in the natural growth condition. The inverse behavior between RNA and protein levels indicates post-transcriptional regulations.

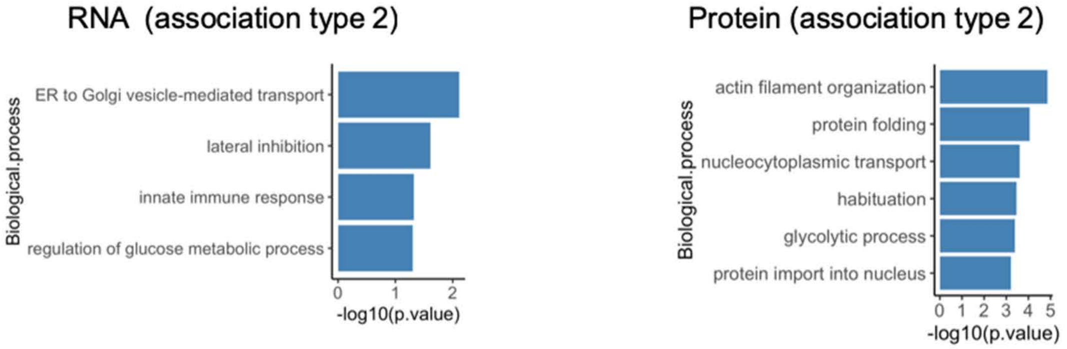

Appendix Figure S6. GO analyses of the associated RNAs and proteins with a type-2 association. GO enrichment analyses on the wing size-associated RNAs and proteins with a type-2 association with wing size traits (absCS and relCS). Biological processes for RNAs and proteins are shown at enrichment significances at nominal p-value < 0.05 (for RNAs) and at FDR < 10% (for proteins).

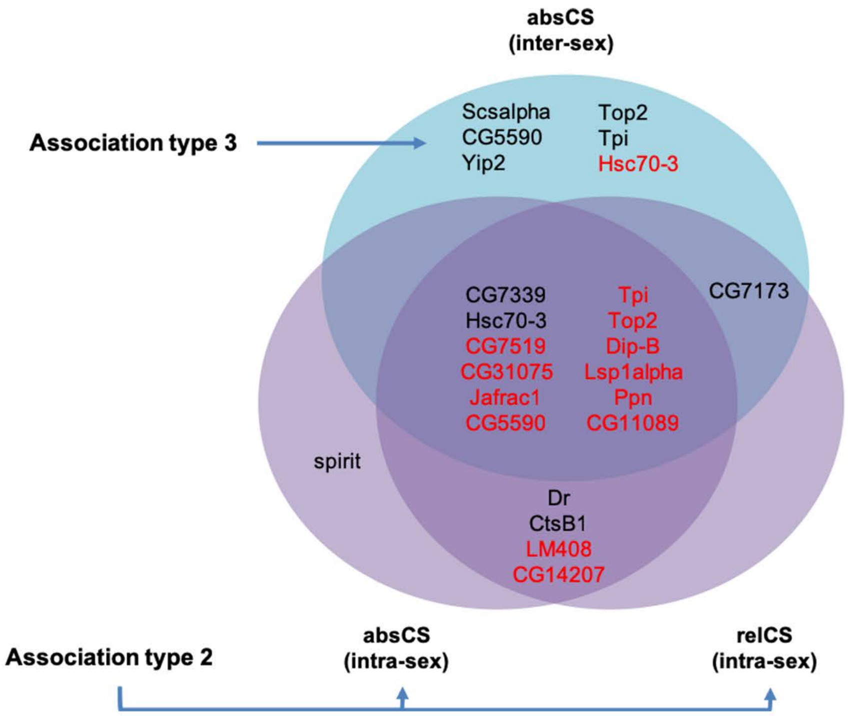

Appendix Figure S7. Association types of growth-regulatory gene candidates tested in the RNAi experiment.

Classification of the association types for the genes tested in the RNAi experiment. Expression levels (RNA and protein) associated with wing size traits are depicted with different colors (black: RNA, red: protein).

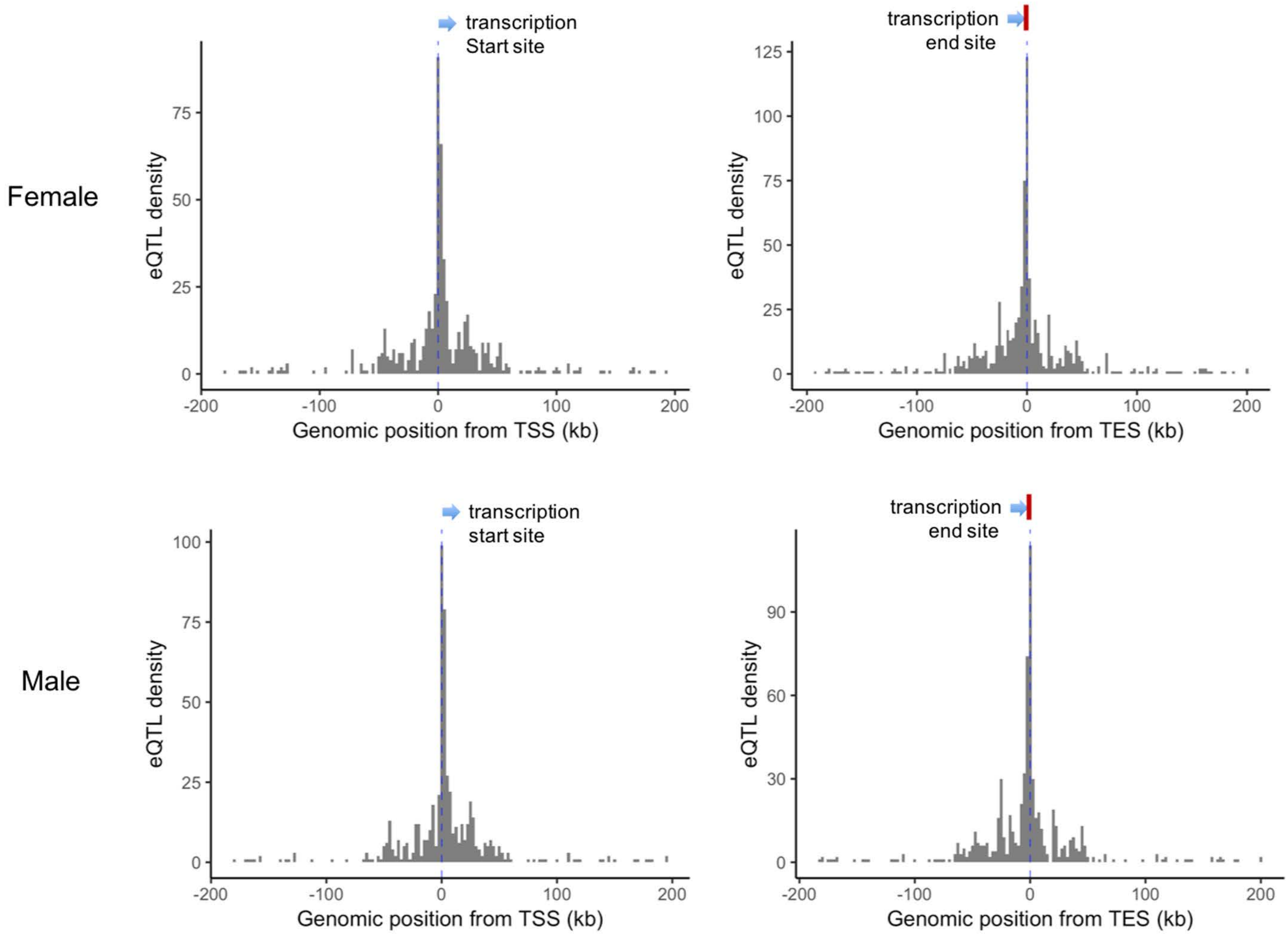

Appendix Figure S8. Predominant localization of eQTL candidates ($p < 1{\times}10^{-8}$) at the TSS and TES. The density of SNPs associated with RNA expression at $p < 1{\times}10^{-8}$ is plotted against the genomic location relative to the start and end sites of transcription (TSS and TES) for each sex separately.

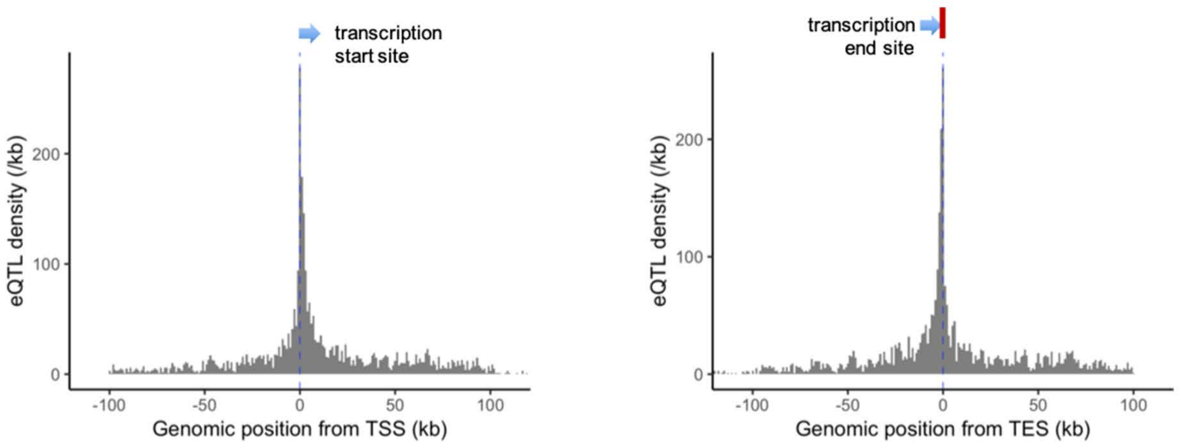

Appendix Figure S9. eQTL density plots for males.
The number of eQTLs per kb is plotted against the genomic location relative to the start and end sites of transcription (TSS and TES) in males.

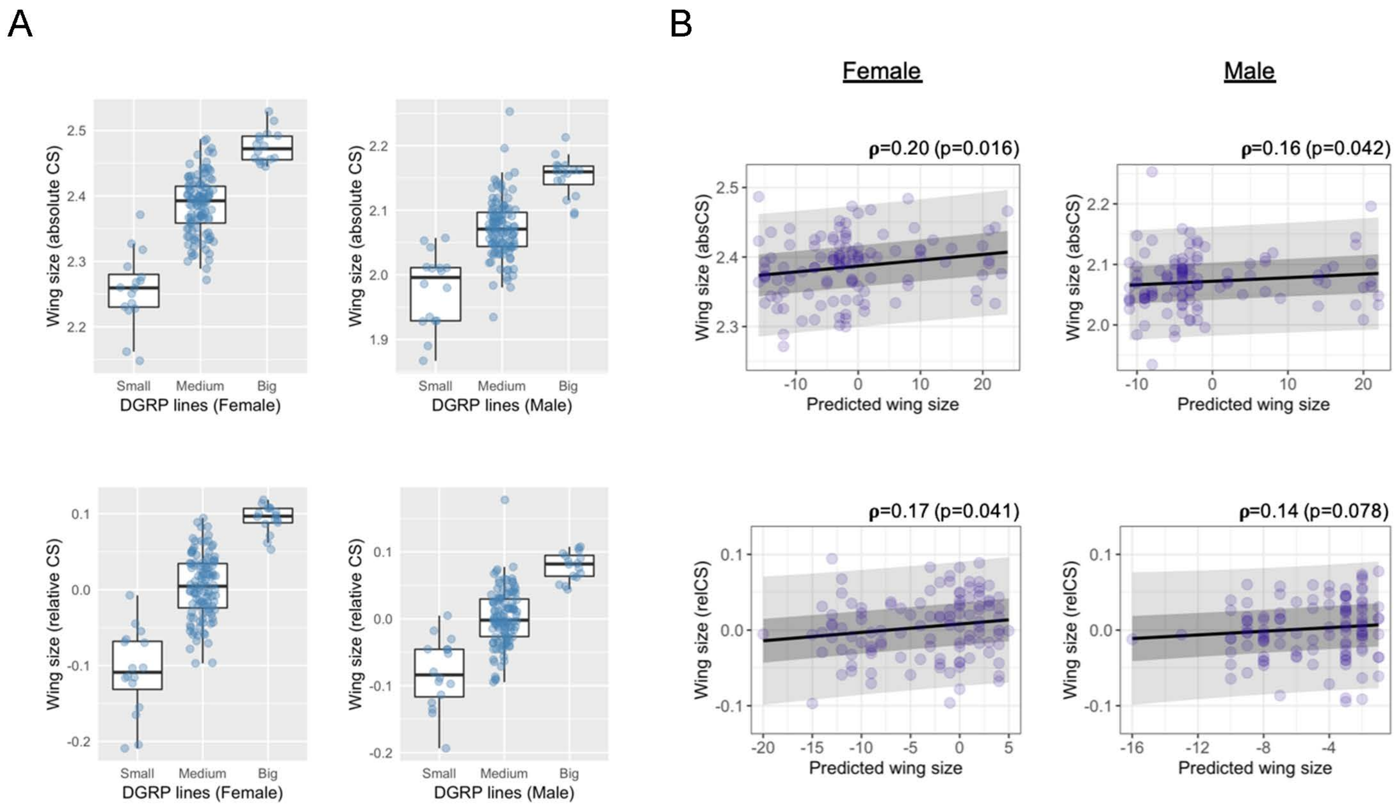

Appendix Figure S10. Wing size prediction of independent DGRP lines with medium-sized wings. (A) Wing size traits (absolute and relative CSs) of DGRP lines are plotted separately for each sex. The 143 DGRP lines for which wing size traits are measured are split into 3 categories based on their wing size (16 small, 111 medium, and 16 big wing lines). The small and big wing lines were used in TWAS to quantify RNA levels in the study. Note that wing size traits of the majority of the 111 medium-sized lines are concentrated around the population means. (B) Wing size prediction of the independent, medium-sized lines based on the mediator-linked genotypes. Spearman correlation coefficients and the P-values are shown for each case (size traits, sexes). The dark and light grey zones indicate 50% and 95% prediction ranges.

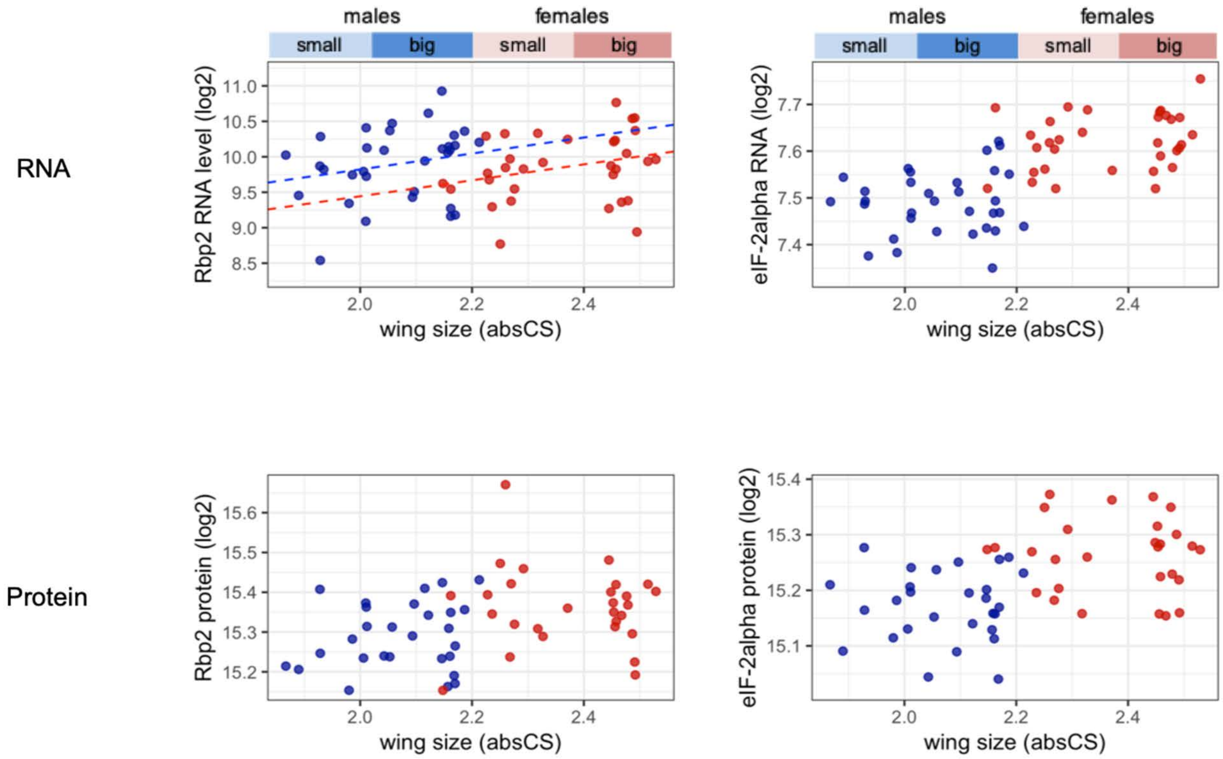

Appendix Figure S11. Expression – trait associations of translation initiation factors that are located next to CG34015.
Plots of RNA and protein levels against wing size are shown for two translation initiation factors Rbp2 and eIF-2alpha neighboring CG34015. Rbp2 shows a weak, type-2 association at the RNA level but a type-3 association at the protein level. eIF-2alpha exhibits strong type-3 associations at both levels.
