# Supplementary material for: Sex‐dependent and sex‐independent regulatory systems of size variation in natural populations
Source: Mol Syst Biol. 2019 Nov 26;15(11):e9012. doi: 10.15252/msb.20199012 (PMC6878047; doi:10.15252/msb.20199012)
Supplement: Supplementary file 2 — Expanded View Figures PDF [file MSB-15-e9012-s002.pdf]

Expanded View Figures

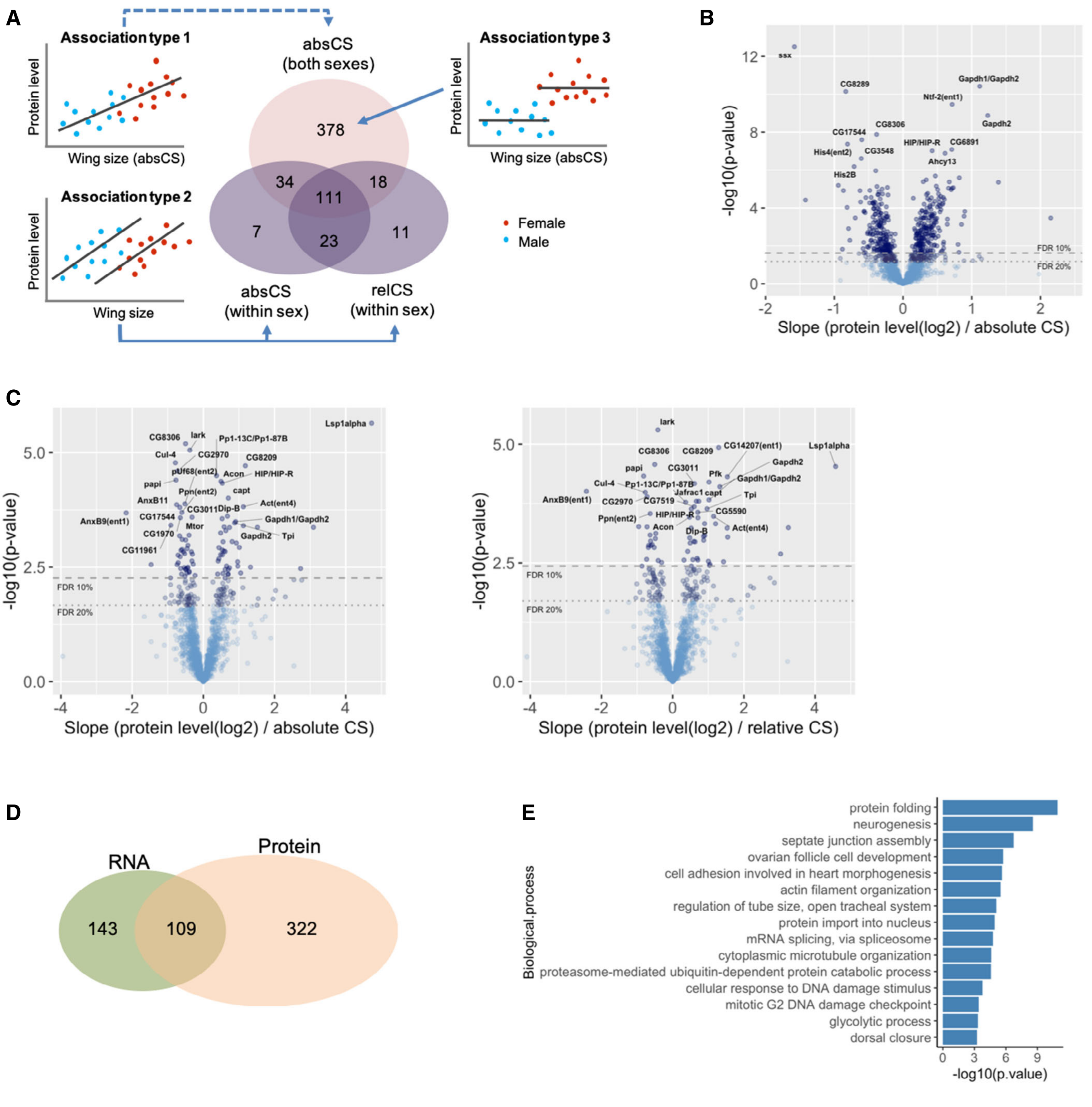

Figure EV1. Trait–protein expression association studies.

A Classification of the wing size-associated proteins at FDR < 20% based on association types. Proteins that exclusively belong to the association type 1 (without overlaps with association type 2) have a type-3 association with wing size.

B Association of proteins with the whole wing size variation that encompasses both sexes. Volcano plot of *P*-values against the slope of the fitted lines. The horizontal lines indicate 10 and 20% FDR thresholds.

C Association of proteins with between-line wing size variation (absolute/relative CS, adjusted for sex). The horizontal lines indicate 10 and 20% FDR thresholds.

D Overlap between size-associated RNAs and proteins. Comparison is made among 1,213 genes that were co-identified at both levels.

E GO enrichment analyses on the wing size-associated proteins (at FDR < 20%). Biological processes associated with wing size at FDR < 0.1 are shown.

© 2019 The Authors

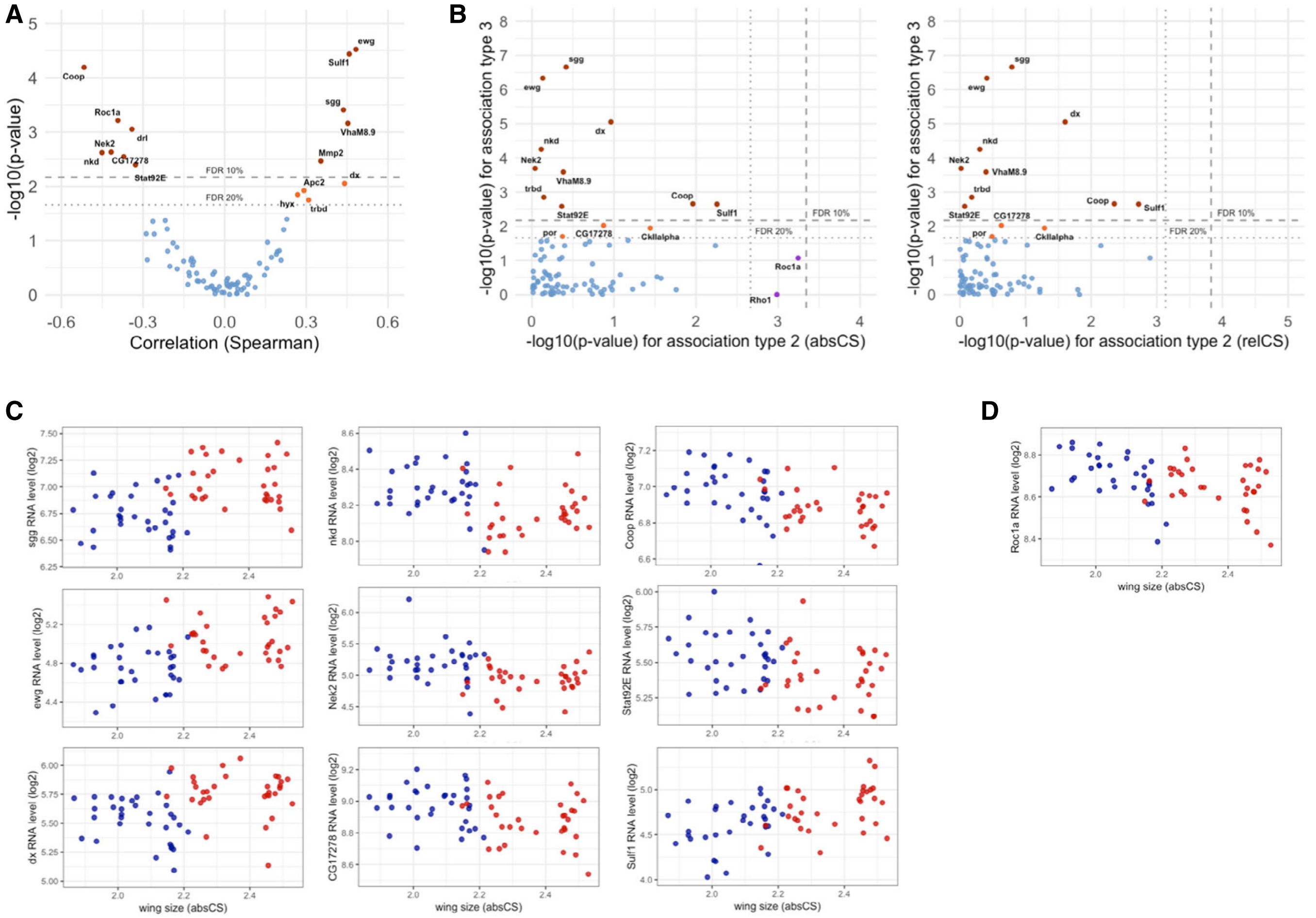

Figure EV2. Pathway components of Wnt signaling are associated with sexual size dimorphism.

A Association of RNA levels from Wnt signaling components with the whole wing size variation that encompasses both sexes. Volcano plot of the *P*-values against Spearman correlation coefficients with wing size is shown. The horizontal lines indicate 10 and 20% FDR thresholds.

B Association of RNA levels from Wnt signaling components with wing size (absCS and relCS). *P*-values from the tests for association type 2 and type 3 are plotted. The dashed and dotted lines indicate 10 and 20% FDR thresholds, respectively. Note that Wnt signaling components mostly exhibit type-3 association.

C Sexually dimorphic expression pattern of Wnt signaling components. Plots of RNA levels confirm type-3 association with wing size.

D An exceptional Wnt signaling component (Roc1a) that is associated with between-line size variation (association type 2).

 © 2019 The Authors

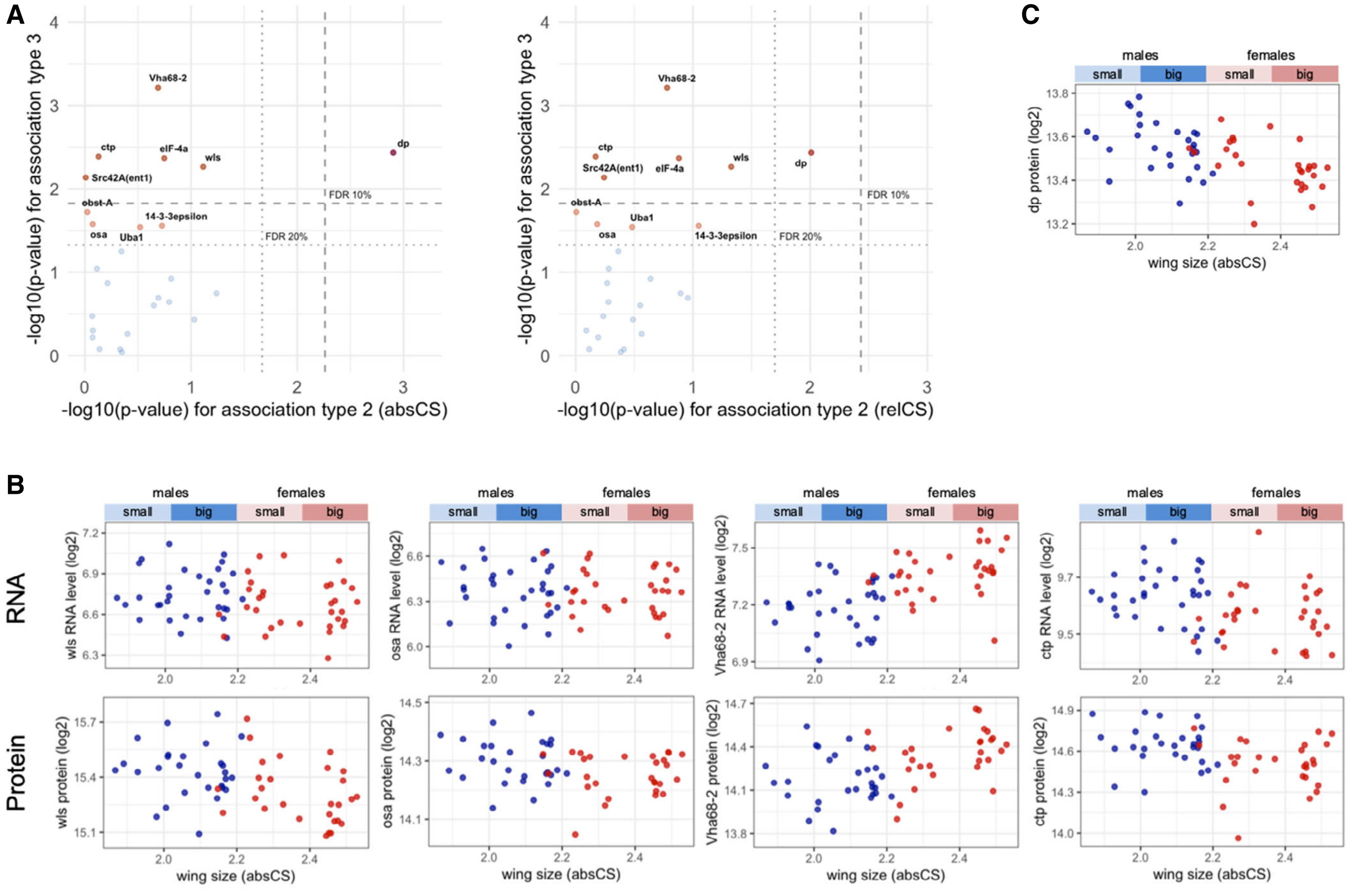

Figure EV3. Protein levels of canonical growth regulators are associated with sexual size dimorphism.

A Association of protein levels from canonical growth regulators with wing size (absCS and relCS). *P*-values from the tests for association type 2 and type 3 are plotted. The dashed and dotted lines indicate 10 and 20% FDR thresholds, respectively. Note that all the associated proteins exhibit type-3 association.

B Sexually dimorphic expression pattern of canonical growth genes at the protein level. Plots of RNA and protein levels against wing size are depicted.

C An exceptional growth protein that is associated with between-line size variation (association type 2).

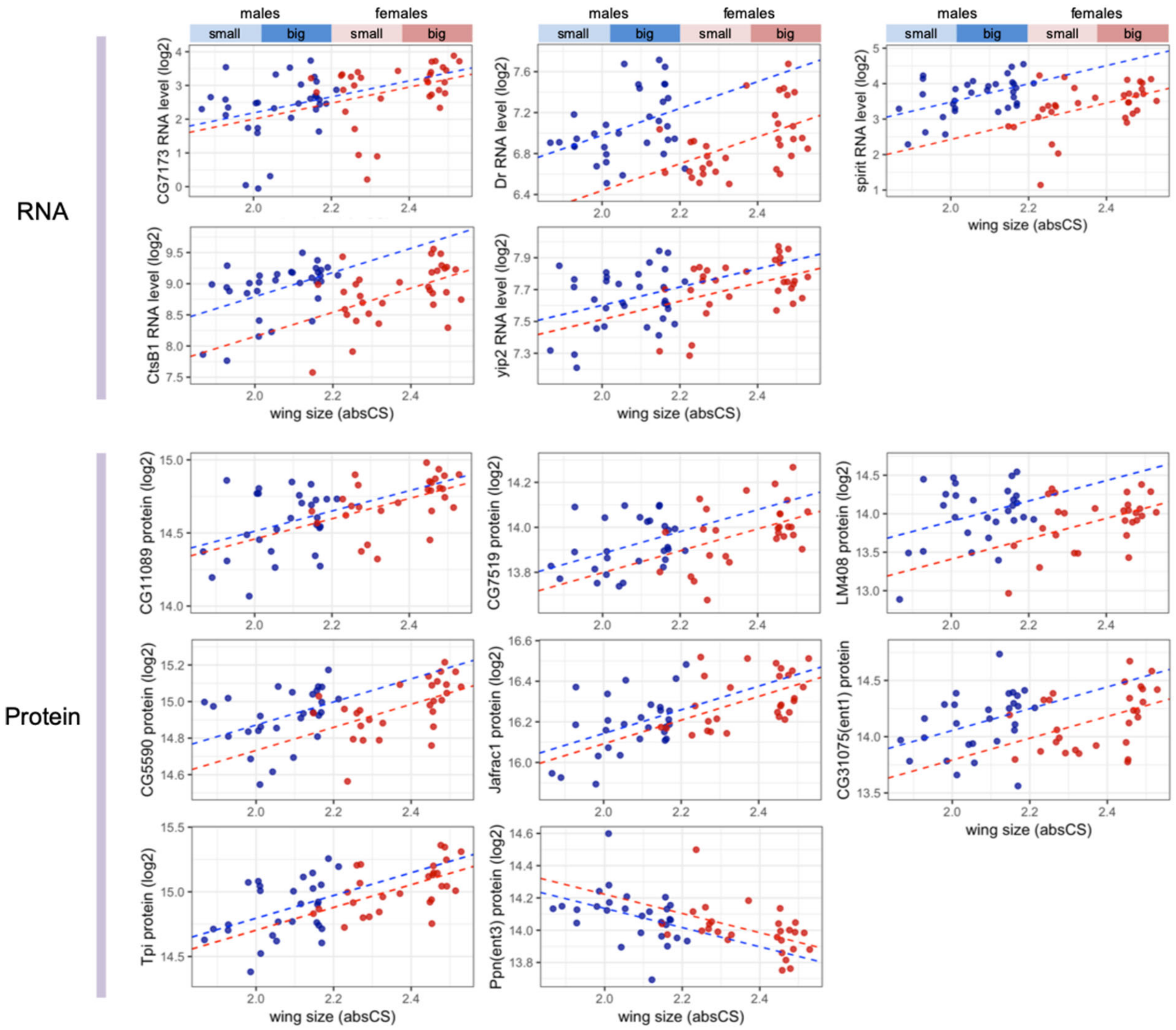

Figure EV4. Trait–expression associations of novel growth-regulatory genes validated in the RNAi experiment.

Expression levels of novel growth genes tested by RNAi show a positive association with wing size in the natural setting except Ppn that exhibits a negative association. Protein levels were preferentially plotted against RNA levels when the protein levels were available. The dotted lines for each sex (red: female, blue: male) are the ones fitted by ANCOVA.

 © 2019 The Authors

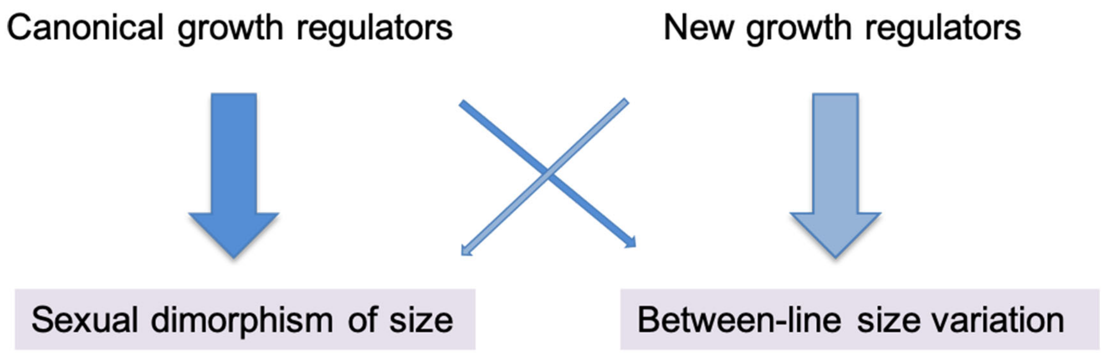

Figure EV5. Working model on the molecular mechanism on wing size control.

Previously identified, canonical growth regulators mainly regulate the sexual dimorphism of wing size. Wing size variation among lines with different sizes within each sex is largely controlled by novel growth regulators identified in the study.

© 2019 The Authors
